# Complex Modified Projective Synchronization of Fractional-Order Complex-Variable Chaotic System with Unknown Complex Parameters

**DOI:** 10.3390/e21040407

**Published:** 2019-04-17

**Authors:** Ruoxun Zhang, Shiwen Feng, Shiping Yang

**Affiliations:** 1 College of Primary Education, Xingtai University, Xingtai 054001, China; 2 College of Physics Science and Information Engineering, Hebei Normal University, Shijiazhuang 050016, China

**Keywords:** complex modified projective synchronization (CMPS), complex modified projective synchronization (CMPS), complex-variable chaotic system, unknown complex parameters

## Abstract

This paper investigates the problem of complex modified projective synchronization (CMPS) of fractional-order complex-variable chaotic systems (FOCCS) with unknown complex parameters. By a complex-variable inequality and a stability theory for fractional-order nonlinear systems, a new scheme is presented for constructing CMPS of FOCCS with unknown complex parameters. The proposed scheme not only provides a new method to analyze fractional-order complex-valued systems but also significantly reduces the complexity of computation and analysis. Theoretical proof and simulation results substantiate the effectiveness of the presented synchronization scheme.

## 1. Introduction

In the past twenty years, the application of fractional calculus has become a focus of attention, since fractional derivatives can more accurately describe the actual physical model. So, it has become an efficient and an excellent tool in physics, mathematical science, chemistry, control engineering, finance, signal processing and other fields [1,2,3,4,5,6]. Meanwhile, chaotic dynamics and synchronization of fractional-order nonlinear systems have aroused tremendous attention of many researchers. Many excellent results have been obtained and some types of synchronization have been presented [7,8,9,10,11,12,13,14,15,16,17,18,19,20,21,22]. In various synchronizations, modified projective synchronization (MPS) refers to the master system and slave system being synchronized to a constant scaling diagonal matrix. In secure communication, this scaling feature extends the binary digital to M-nary digital for faster transmission. It is quite clear that complete synchronization (CS), ant-synchronization (AS) and projective synchronization (PS) are each special cases of MPS. Therefore, the study of MPS has important theoretical significance and application prospect.

The above-mentioned works mainly investigated the fractional-order systems with real variables, not involving complex variables. It is well known that complex variables, which double the number of variables, can generate complicated dynamical behaviors, enhance anti-attack ability and achieve higher transmission efficiency [23,24,25]. The complex-variable systems can be widely applied to describe a variety of physical phenomena, for example, atomic polarization amplitudes, electric fields, population inversion [26,27], detuned laser systems, amplitudes of electromagnetic fields, thermal convections of liquid flows [28,29,30], etc. Many researchers have taken included complex into variables in fractional-order systems and have investigated dynamic behavior, stability, stabilization and synchronization of fractional-order complex-variable chaotic systems (FOCCS) in recent years [31,32,33,34,35,36,37]. Especially, as a new synchronization phenomenon, the complex modified projective synchronization (CMPS) was first introduced by Mahmoud et al. [38], which can be seen as an extension of MPS. That is, in complex space, two complex-variable systems can achieve MPS with desired complex scaling factors. As the complex scaling factor is arbitrary and more unpredictable than the real scaling factor, the capacity of the transmitted message is doubled and the safety is greatly strengthened [39]. So, study on CMPS of fractional complex-variable chaotic systems is of great theoretical and practical significance. However, in the existing literature, parameters of the FOCCS are real and known in priori. In fact, in many practical engineering situations, most of system parameters cannot be accurately determined in advance and chaos synchronization will be destroyed with these uncertain factors. Meanwhile, the complex parameter is more complex and unpredictable than the real parameter, and if the initial values are real numbers, the existing FOCCS [24,31,32] will become a fractional-order real-variable chaotic system. Hence, it is an important problem to realize CMPS of FOCCS with unknown complex parameters.

Inspired by the above discussions, the CMPS problem of FOCCS with unknown complex parameters is investigated in this paper. First, we present a stability theory for fractional-order uncertain nonlinear systems. Then, using this theory, the inequality proposed by Xu et al. [39] and complex analysis techniques, we realize CMPS of such systems via constructing a suitable response system. It should be noted that we deal with the synchronization problem of fractional-order uncertain complex-variable systems in the complex domain. That is to say, it is not necessary to separate the complex-variable system into its real and imaginary parts. This greatly reduces the complexity of computation and the difficulty of theoretical analysis.

Notation: ${\mathbb{C}}^{n}$ denotes complex *n*-dimensional space. $z\in {\mathbb{C}}^{n}$, $z^{r},z^{i},\bar{z}$, $z^{T}$, $z^{H}$ and ||z|| represent the real part, imaginary part, conjugate, transpose, conjugate transpose and *l*_2_-norm of z, respectively. For a matrix $A\in {\mathbb{C}}^{n\times n}$, $A^{H}$ denotes its conjugate transpose. ${ℍ}^{n\times n}$ denotes the set of n×n Hermite matrices.

## 2. Preliminaries

### 2.1. Fractional Calculus

**Definition** **1** [40]. *The fractional integral of order α for a function f is defined as*

(1) Iαf(t)=Dt0t −αf(t)=1Γ(α)∫t0t(t−τ)α−1f(τ)dτ

*where t ≥ t_0_ and α > 0.*

**Definition** **2** [40]. *Caputo’s fractional derivative of order α for a function $f\in {\mathbb{R}}^{n}$ is defined by*

(2) Dt0Ctαf(t)=1Γ(n−α)∫t0tf(n)(τ)(t−τ)α−n+1dτ

*where t ≥ t_0_ and n is a positive integer such that n−1<α<1.*

**Lemma** **1.** [41] *Let $x(t)\in {\mathbb{R}}^{n}$ be a continuous and derivable vector function. Then, for any time instant t ≥ t_0_ and α∈(0,1),*

(3) 12Dt0Ctα[xT(t)x(t)]≤xT(t)Dt0Ctαx(t).

**Corollary** **1.** *For a scalar derivable function φ(t) and a constants C, we have*

(4) 12Dt0Ctα(φ(t)−C)2≤(φ(t)−C)Dt0Ctαφ(t).

**Lemma** **2.** [42] *Let $z\;\in {\mathbb{C}}^{n}$ be a differentiable complex-valued vector. Then, $\forall t\geq t_{0}$ and α∈(0,1], the following inequality holds*

(5) Dt0CtαzH(t)Pz(t)≤zH(t)PDt0Ctαz(t)+(Dt0Ctαz(t))HPz(t).

*where $P\in {\mathbb{C}}^{n\times n}$ is a constant positive definite Hermitian matrix.*

**Lemma** **3.** *(Stability theory for fractional-order system) Let $V_{1}(t)$ be a uniformly continuous and derivable Lyapunov function, $V_{2}(t)$ be a derivable and nonnegative function. If*

(6) $$V(t)=V_{1}(t)+V_{2}(t)$$

*and*

(7) D0CtαV(t)≤−θV1(t)

*where θ is a positive constant. Then $\underset{t\to \infty }{\lim}V_{1}(t)=0$.*

**Proof.**  By α-integrating (7), we have:

(8) $$V(t)-V(0)\leq -\theta I^{\alpha }V_{1}(t)\leq 0$$

Thus, V(t)≤V(0), t≥0. From (6), we can obtain that $V_{1}(t)$ and $V_{2}(t)$ are bounded.

Next, we adopt contradiction to prove $\underset{t\to \infty }{\lim}V_{1}(t)=0$ using the idea of Theorem 1 in [43].

Suppose that $V_{1}(t)\neq 0$ as t→∞. Then there exists a monotone increasing sequence ${\left(t_{k}\right)}_{k\in N^{\;+}}$ ($t_{k}\to \infty$ as k→∞) and a positive constant ε > 0 such that $V_{1}(t_{k})>\varepsilon$. Since the uniform continuity of $V_{1}(t)$, for the given ε, $\exists \delta >0\;\;(\delta \leq \inf_{j\in N^{+}}\{t_{j+1}-t_{j}\},$ which implies that the intervals $\left[t_{k},t_{k}+\delta \right]$ are nonoverlapping) such that $|V_{1}(t_{k})-V_{1}(t)|\leq \varepsilon /2.$ Then, for any $t\in [t_{k},t_{k}+\delta ]$ it follows that $V_{1}(t)=\;V_{1}(t_{k})-V_{1}(t_{k})+V_{1}(t)$ $\geq \;|V_{1}(t_{k})|-|V_{1}(t_{k})-V_{1}(t)|\;>\varepsilon /2$. Thus, for any *k* = 1, 2, 3, …, from (7), we have:

$$\begin{array}{ll} V(t_{k}+\delta )-V(0) & \leq \frac{-\theta }{\Gamma (\alpha )}\int_{0}^{t_{k}+\delta }\frac{V_{1}(\tau )}{{\left(t_{k}+\delta -\tau \right)}^{1-\alpha }}d\tau \;\\ & =\frac{-\theta }{\Gamma (\alpha )}[\int_{0}^{t_{1}}+\;\int_{t_{1}}^{t_{1}+\delta }+\;\int_{t_{1}+\delta }^{t_{2}}+\;\int_{t_{2}}^{t_{2}+\delta }+\int_{t_{2}+\delta }^{t_{3}}+\;\int_{t_{3}}^{t_{3}+\delta }+\;\cdots \;+\int_{t_{k}}^{t_{k}+\delta }]\;\;\frac{V_{1}(\tau )}{{\left(t_{k}+\delta -\tau \right)}^{1-\alpha }}d\tau \\ & \leq \frac{-\theta }{\Gamma (\alpha )}[\int_{t_{1}}^{t_{1}+\delta }+\int_{t_{2}}^{t_{2}+\delta }+\;\;\int_{t_{3}}^{t_{3}+\delta }+\;\cdots +\int_{t_{k}}^{t_{k}+\delta }]\;\;\frac{V_{1}(\tau )}{{\left(t_{k}+\delta -\tau \right)}^{1-\alpha }}d\tau \\ & \leq \frac{-\theta \varepsilon }{2\Gamma (\alpha )}[\int_{t_{1}}^{t_{1}+\delta }+\int_{t_{2}}^{t_{2}+\delta }+\;\;\int_{t_{3}}^{t_{3}+\delta }+\;\cdots +\int_{t_{k}}^{t_{k}+\delta }]\;\;{\left(t_{k}+\delta -\tau \right)}^{\alpha -1}d\tau \\ & =\frac{-\theta \varepsilon }{2\Gamma (\alpha )}\sum_{j=1}^{k}\int_{t_{j}}^{t_{j}+\delta }\;{\left(t_{k}+\delta -\tau \right)}^{\alpha -1}\;d\tau \end{array}$$

Given that ${\left(t_{k}+\delta -\tau \right)}^{\alpha -1}\geq {\left(t_{k}+\delta -t_{j}\right)}^{\alpha -1}$ for all $\tau \in [t_{j},\;t_{j}+\delta ]$ results in:

$$\begin{array}{ll} V(t_{k}+\delta )-V(0) & \leq \frac{-\theta \varepsilon }{2\Gamma (\alpha )}\sum_{j=1}^{k}\int_{t_{j}}^{t_{j}+\delta }\;{\left(t_{k}+\delta -\tau \right)}^{\alpha -1}\;d\tau \leq \frac{-\theta \varepsilon }{2\Gamma (\alpha )}\sum_{j=1}^{k}\int_{t_{j}}^{t_{j}+\delta }\;{\left(t_{k}+\delta -t_{j}\right)}^{\alpha -1}\;d\tau \\ & =\frac{-\theta \varepsilon \delta }{2\Gamma (\alpha )}\sum_{j=1}^{k}{\left(t_{k}+\delta -t_{j}\right)}^{\alpha -1}\;\leq \frac{-\theta \varepsilon \delta }{2\Gamma (\alpha )}\sum_{j=1}^{k}{\left(t_{k}+\delta -t_{1}\right)}^{\alpha -1}\\ & =\frac{-\theta \varepsilon \delta }{2\Gamma (\alpha )}\frac{k}{{\left(t_{k}+\delta -t_{1}\right)}^{1-\alpha }}\;\leq \frac{-\theta \varepsilon \delta }{2\Gamma (\alpha )}\;\frac{k}{{\left(kd\right)}^{1-\alpha }}\;=\frac{-\theta \varepsilon \delta }{2\Gamma (\alpha )}\;\frac{k^{\alpha }}{{\left(d\right)}^{1-\alpha }} \end{array}$$

where $d=\sup_{j\in N,\;2\;\leq j\;\leq k}\{t_{j}-t_{j-1}\}$ (since $V_{1}(t)$ is a uniformly continuous function and assumed $V_{1}(t)\neq 0$, as t→∞, *d* is bounded). Obviously, $V(t_{k}+\delta )-V(0)\leq \frac{-\theta \varepsilon \delta }{2\Gamma (\alpha )}\;\frac{k^{\alpha }}{{\left(d\right)}^{1-\alpha }}\to -\infty$ as k→∞, which contradicts with V(t)≥0. Therefore, $\underset{t\to \infty }{\lim}V_{1}(t)=0$.  ☐

**Remark** **1.** *Lemma 3 provides a stability criterion for the fractional-order nonlinear uncertain system by choosing a Lyapunov function that includes two parts V_1_(t) and V_2_(t).*

**Remark** **2.** *Lemma 3 provides a Lyapunov-based adaptive control method for stability analysis and synchronization of fractional-order systems (FOS).*

**Remark** **3.** *Lemma 3 is suitable for verifying the stability and stabilization controller design of FOS with unknown parameters and external disturbances.*

**Remark** **4.** *If the FOS has no uncertainties, then Lemma 3 is still valid.*

**Lemma** **4.** [44] *For the fractional-order complex-variable systems*

(9) D0Ctαz(t)=h(z(t))

*where 0<α<1, $z(t)={\left(z_{1},z_{2},\cdots ,z_{n}\right)}^{T}\in {\mathbb{C}}^{n}$ is the system complex state vector, $h\in {\mathbb{C}}^{n}$ is a continuous nonlinear function complex vector, which satisfies the globally Lipschitz continuity condition in the complex domain. Let z(t)=0 be an equilibrium point of system (1), and let $V_{1}(t)=z^{H}(t)z(t)$ and $V_{2}(z(t))\geq 0$ are continuously differentiable functions. If*

$$V(t)=V_{1}(t)+V_{2}(z(t))$$

*and*

D0ttαV(t)≤−θV1(t)

*where θ is a positive constant, then z(t) = 0 is asymptotically stable.*

**Lemma** **5.** [45,46] *For all $z\in {\mathbb{C}}^{n}$, $B\in H^{n\times n}$, there exists a positive constant l_1_ such that the following inequality holds:*

$$\lambda_{\min}(B)z^{H}z\leq z^{H}Bz\leq \lambda_{\max}(B)z^{H}z$$

*where $\lambda_{\min}$ and $\lambda_{\max}$ are the minimum and maximum eigenvalue of B.*

**Lemma** **6.** [44,46] *For the Lipschitz continuous function $f\in {\mathbb{C}}^{n}$, there exists a positive constant l_2_ such that the following inequality holds:*

(10) $${\left(z-w\right)}^{H}[f(z)-f(w)]+{\left[f(z)-f(w)\right]}^{H}(z-w)\leq l_{2}{\left(z-w\right)}^{H}(z-w)$$

### 2.2. System Model

We consider a kind of fractional-order complex-variable chaotic drive and response systems

(11) D0Ctαz(t)=Fz(t)+f(z(t))

(12) D0Ctαw(t)=Hw(t)+h(w(t))

where 0<α<1, $z(t)={\left(z_{1},z_{2},\cdots ,z_{n}\right)}^{T}\in {\mathbb{C}}^{n}$ and $w(t)={\left(w_{1},w_{2},\cdots ,w_{n}\right)}^{T}\in {\mathbb{C}}^{n}$ are the system complex state vectors, $f\in {\mathbb{C}}^{n}$ and $h\in {\mathbb{C}}^{n}$ are continuous complex nonlinear function vectors, $F\in {\mathbb{C}}^{n\times n}$ and $H\in {\mathbb{C}}^{n\times n}$ are complex or real parameter matrices.

**Definition** **3.** [38] *For given drive system (8) and response system (9), it is said to achieve CMPS if*

$$\underset{t\to \infty }{\lim}||w(t)-Mz(t)||=0$$

*where $M\in {\mathbb{C}}^{n\times n}$ is the desired scaling diagonal complex matrix.*

**Remark** **5.** *Obviously, some known synchronization ways are the special cases of CMPS, such as CS, AS, PS and MPS.*

## 3. Main Results

We consider a kind of FOCCS with unknown complex parameters as

(13) D0Ctαz(t)=Az(t)+f(z(t))=g(z(t))θ+f(z(t))

where 0<α<1, $z(t)={\left(z_{1},z_{2},\cdots ,z_{n}\right)}^{T}\in {\mathbb{C}}^{n}$ is the system complex state vector, $f\in {\mathbb{C}}^{n}$ is a continuous nonlinear function vector, which satisfies the globally Lipschitz continuity condition in the complex domain, $A\in {\mathbb{C}}^{n\times n}$ is an unknown (complex or real) parameter matrix and $g\in {\mathbb{C}}^{n\times m}$ is a complex function matrix, and $\theta ={\left(\theta_{1},\theta_{2},\cdot \cdot \cdot ,\theta_{m}\right)}^{T}$ is the system unknown complex parameter vector. 

System (13) was chosen as the master system. In this case, we constructed the slave system as follows:

(14) D0Ctαw(t)=Mg(M−1w(t))θ^+Mf(M−1w(t))+u(t)

where $w(t)={\left(w_{1},w_{2},\cdots ,w_{n}\right)}^{T}$ is the complex state vector, $\hat{\theta }\in {\mathbb{C}}^{m}$ represents the estimate vector of the unknown vector θ, $M\in {\mathbb{C}}^{n\times n}$ is the desired scaling diagonal complex matrix, and $u(t)={\left(u_{1}(t),u_{2}(t),\cdots ,u_{n}(t)\right)}^{T}$ is the controller, to be determined.

Remark 2. From (13), it follows that g(z(t))θ=Az(t) and $g(M^{-1}w(t))\theta =AM^{-1}w(t)$.

**Theorem** **1.** *Asymptotically CMPS of systems (13) and (14) can be achieved under adaptive controller*

(15) u(t)=−ke(t)

*and the complex update laws:*

(16) D0Ctαk=σeH(t)e(t) 

(17) D0Ctαeθ=D0Ctαθ^=−ηgH(M−1w(t))MHe(t)

*where e(t)=w(t)−Mz(t) is the error vector, $e_{\theta }=\hat{\theta }-\theta$ is the parameter error, θ,η are two arbitrary positive constants.*

**Proof.**  From the error vector and systems (13) and (14), it follows:

(18) D0Ctαe(t)=D0Ctαw(t)−MD0Ctαz(t)=Mg(M−1w(t))θ^+Mf(M−1w(t))−M[g(z(t))θ+f(z(t))]−ke(t)=Mg(M−1w(t))(θ^−θ)+M[g(M−1w(t))−g(z(t))]θ+M[f(M−1w(t))−f(z(t))]−ke(t)=Mg(M−1w(t))eθ+M[g(M−1w(t))−g(z(t))]θ+M[f(M−1w(t))−f(z(t))]−ke(t)

Let us present the Lyapunov function:

(19) $$V(t)=V_{1}(t)+V_{2}(t)$$

where $V_{1}(t)=e^{H}(t)e(t),\;V_{2}(t)=\frac{1}{\sigma }{\left(k-k^{*}\right)}^{2}+\frac{1}{\eta }e_{\theta }^{H}(t)e_{\theta }(t)$, $k^{*}$ is to be determined.

Using Lemma 1, Corollary 1 and Lemma 2, we have:

D0CtαV(t)=D0Ctα[eH(t)e(t)+1σ(k−k*)2+1ηeθH(t)eθ(t)]≤eH(t)D0Ctαe(t)+[D0Ctαe(t)]He(t)+2σ(k−k*)D0Ctαk+1ηeθH(t)D0Ctαeθ(t)+1η[D0Ctαeθ(t)]Heθ(t)≤eH(t){Mg(M−1w(t))eθ+M[g(M−1w(t))−g(z(t))]θ+M[f(M−1w(t))−f(z(t))]−ke(t)}+{Mg(M−1w(t))eθ+M[g(M−1w(t))−g(z(t))]θ+M[f(M−1w(t))−f(z(t))]−ke(t)}He(t)+2σ(k−k*)D0Ctαk+1ηeθH(t)D0Ctαeθ(t)+1η[D0Ctαeθ(t)]Heθ(t)

Substituting Equations (16) and(17) into the inequality above, we further have:

D0CtαV(t)≤eH(t){Mg(M−1w(t))eθ+M[g(M−1w(t))−g(z(t))]θ+M[f(M−1w(t))−f(z(t))]−ke(t)}+{Mg(M−1w(t))eθ+M[g(M−1w(t))−g(z(t))]θ+M[f(M−1w(t))−f(z(t))]−ke(t)}He(t)+2(k−k*)eH(t)e(t)−eθH(t)gH(w(t))e(t)−[gH(w(t))e(t)]Heθ(t)

$$\begin{array}{l} =e^{H}(t)MA[M^{-1}w(t)-z(t)]+{\left[M^{-1}w(t)-z(t)\right]}^{H}A^{H}M^{H}e(t)\\ +e^{H}(t)M[f(M^{-1}w(t))-f(z(t))]+{\left[f(M^{-1}w(t))-f(z(t))\right]}^{H}M^{H}e(t)-2k^{*}e^{H}(t)e(t) \end{array}$$

From Lemma 4 and Lemma 5, we can obtain:

$$\begin{array}{l} e^{H}(t)MA[M^{-1}w(t)-z(t)]+{\left[M^{-1}w(t)-z(t)\right]}^{H}A^{H}M^{H}e(t)\\ =e^{H}(t)MAM^{-1}e(t)+e^{H}(t){\left(M^{-1}\right)}^{H}A^{H}M^{H}e(t)\\ =e^{H}(t)[MAM^{-1}+{\left(M^{-1}\right)}^{H}A^{H}M^{H}]e(t)\leq L_{1}e^{H}(t)e(t) \end{array}$$

and

$$\begin{array}{l} e^{H}(t)M[f(M^{-1}w(t))-f(z(t))]+{\left[f(M^{-1}w(t))-f(z(t))\right]}^{H}M^{H}e(t)\\ ={\left[M^{-1}w(t)-z(t)\right]}^{H}M^{H}M[f(M^{-1}w(t))-f(z(t))]\\ \;\;+{\left[f(M^{-1}w(t))-f(z(t))\right]}^{H}M^{H}M[M^{-1}w(t)-z(t)]\\ \leq l_{2}{\left[M^{-1}w(t)-z(t)\right]}^{H}[M^{-1}w(t)-z(t)]\\ \leq l_{2}e^{H}(t){\left(M^{-1}\right)}^{H}M^{-1}e(t)\leq L_{2}e^{H}(t)e(t) \end{array}$$

where *L*_1_, *l*_2_ and *L*_2_ are three positive constants. Then, one has:

D0CtαV(t)≤(L1+L2−2k*)eH(t)e(t)

Let $2k^{*}=L_{1}+L_{2}+1$, then:

(20) D0CtαV(t) ≤−eH(t)e(t)=−V1(t).

By α-integrating (20), we have V(t)≤V(0), which implies $e^{H}(t)e(t),||e_{\theta }||,||k(t)||$ are bounded and ||D0Ctαe(t)|| is also bounded according to (18) and system (13) being chaotic. Then, from [44], $V_{1}(t)=e^{H}(t)e(t)$ is uniformly continuous. Therefore, it follows from (19) and (20) and Lemma 3 that

$$\underset{t\to \infty }{\lim}V_{1}(t)=\underset{t\to \infty }{\lim}e^{H}(t)e(t)=0.$$

Therefore, the systems (13) and (14) can reach asymptotically CMPS under the adaptive control strategy (15–17). ☐

**Remark** **6.** *Unlike previous works, in our proposed method, the entire analysis process is performed in the complex-valued domain, and the complex function theory is used to derive synchronization conditions without separating the original complex-valued chaotic system into two real-valued systems, which reduces the complexity of analysis and computation.*

**Remark** **7.** *If the system parameters are known, the update law will be reduced to (16) only.*

## 4. Numerical Simulations

In this section, in order to show the effectiveness of the proposed scheme in preceding section, numerical example on fractional-order complex chaotic system will be provided. When numerically solving such systems, we adopted the Grünwald–Letnikov (G-L) method [47] using MATLAB. Lyapunov exponents of the systems were calculated by adopting the Wolf et al. algorithm [48] with some changes.

**Figure 1:**
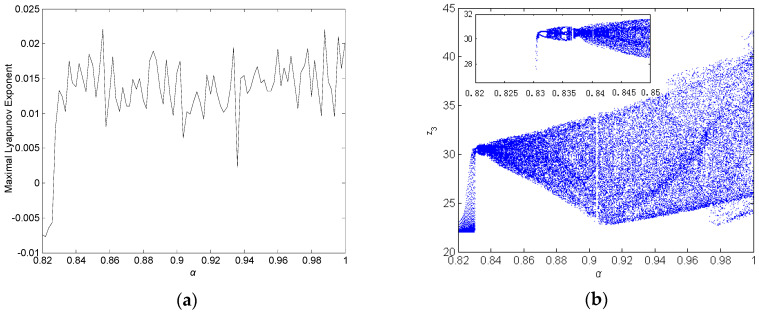
Dynamic behavior of fractional-order complex Chen system with commensurate order α(a=30+i, b=3, c=26+0.6i). (**a**) Maximal Lyapunov exponent spectrum. (**b**) The bifurcation diagram.

**Figure 2:**
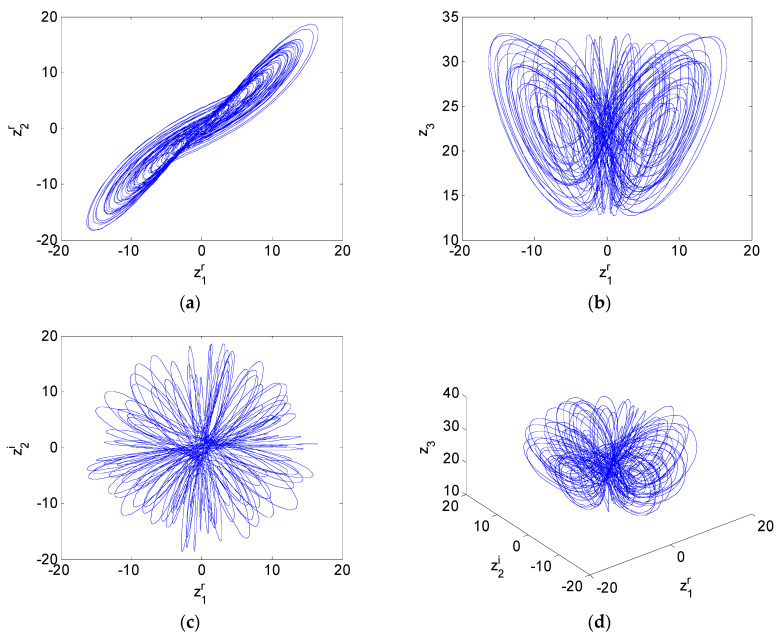
Chaotic attractors of fractional-order complex Chen system with a=30+i, b=3, c=26+0.6i, α=0.9. (**a**) $z_{1}^{r}-z_{2}^{r}$, (**b**) $z_{1}^{r}-z_{3}$, (**c**) $z_{1}^{r}-z_{2}^{i}$, (**d**) $z_{1}^{r}-z_{2}^{i}-z_{3}$.

**Figure 3:**
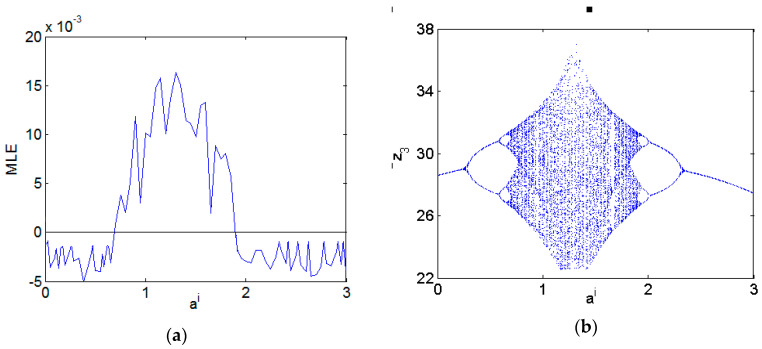
Dynamic behaviors of the fractional-order complex Chen System with varying *a^i^* ($a^{r}=30+i,\;b=3,\;c=26+0.6i,\;\alpha =0.95$). (**a**) Maximal Lyapunov Exponent. (**b**) Bifurcation diagram.

Consider the fractional-order Chen complex chaotic system with commensurate order:

(21) (D0Ctαz1D0Ctαz2D0Ctαz3)=(a(z2−z1)(c−a)z1+cz2−z1z3(z¯1z2+z1z¯2)/2−bz3)=(z2+z100−z1z1+z2000−z3)(acb)+(0−z1z3(z¯1z2+z1z¯2)/2)

where $z_{1},z_{2},z_{3}$ are the complex state variables and *a, b, c* are system parameters, let a=30+i,  b=3, c=26+0.6i. The maximum Lyapunov exponent (MLE) spectrum is depicted in Figure 1a, and the bifurcation diagram is presented in Figure 1b. Figure 1a,b show that system (19) is chaotic with fractional order α∈[0.832,1] and parameters a=30+i, b=3, c=26+0.6i. When the fractional-order α=0.9, the attractor trajectories are illustrated in Figure 2. Recently, [49] described how to perform a successful simulation and optimization, and how to synthesize the mathematical models using CMOS technology. The application of metaheuristics to optimize MLE by varying the parameters of the oscillators was discussed. Here, the MLE spectrum with varying parameter *a^i^* (the imaginary of *a*) is depicted in Figure 3a, the bifurcation diagram is presented in Figure 3b.

Assuming the parameters a,b,c are unknown, we select the complex scaling matrix $M=\left(\begin{array}{ccc} 1+i & 0 & 0\\ 0 & 1-i & 0\\ 0 & 0 & 1 \end{array}\right)$. According to Theorem 1, we construct the response system as follows:

(22) (D0Ctαw1D0Ctαw2D0Ctαw3)=(w2​ i−w100w1 iw2−w1 i000−w3)(a^c^b^)+(0w1w3 i(w¯1w2−w1w¯2)i/2)+(u1u2u3)

where $\hat{a},\hat{b},\hat{c}$ are parameter estimations. $u_{1},u_{2},u_{3}$ are the controllers. 

The error vector $e={\left(\begin{array}{ccc} e_{1} & e_{2} & e_{3} \end{array}\right)}^{T}=z-Mw=\left(\begin{array}{c} z_{1}-(1+i)w_{1}\\ z_{2}-(1-i)w_{2}\\ z_{3}-w_{3} \end{array}\right)$ According to Theorem 1, the controllers and the update rules are selected as:

(23) u1=−ke1, u2=−ke2, u3=−ke3D0Ctαk=σeHe=σ(e¯1e1+e¯2e2+e¯3e3), (σ>0)

(24) (D0Ctαa^D0Ctαc^D0Ctαb^)=−(w2i−w100w1iw2−w1i000−w3)H(e1e2e3)=((w¯2i+w¯1)e1+w¯1ie2−(w¯2+w¯1i)e2w¯3e3)

**Figure 4:**
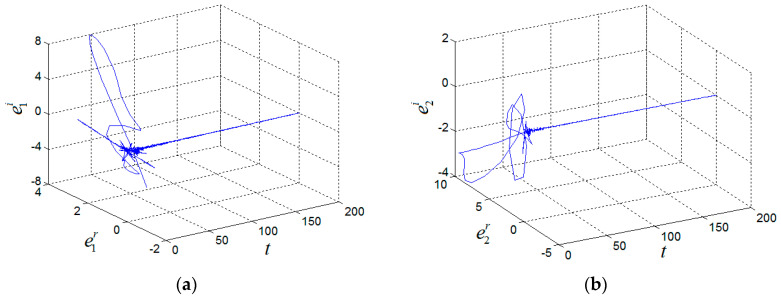

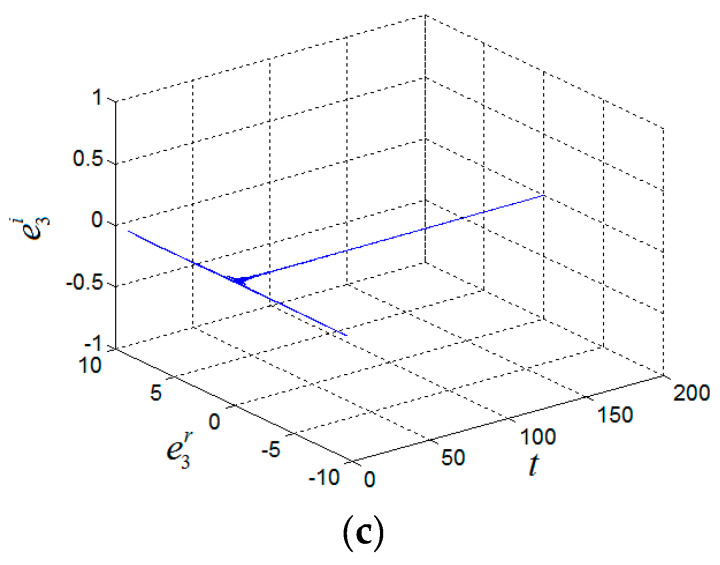
CMPS errors of the fractional-order complex Chen System. (**a**) $e_{1}$, (**b**) $e_{2}$, (**c**) $e_{3}$.

**Figure 5:**
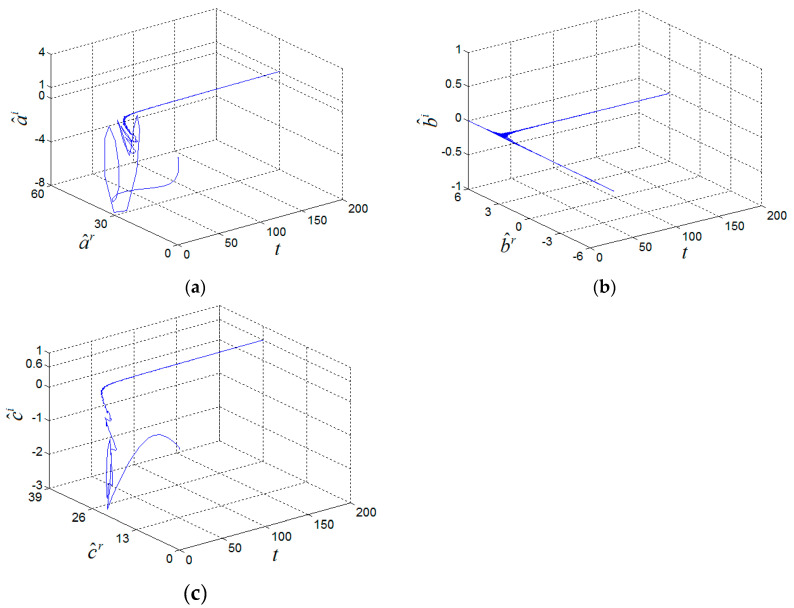
Estimated complex parameters of the fractional-order complex Chen System, (**a**) $\hat{a}$, (**b**) $\hat{b}$, (**c**) $\hat{c}$.

In the simulation, let α=0.9, (***a*, *b***, *c*) = (30 + *i*, 3, 26 + 0.6*i*), the initial conditions z(0)= ${\left(1+i,-2-i,6\right)}^{T}$, $w(0)={\left(-1+i,-3+i,10\right)}^{T}$, $\left(\hat{a}(0),\hat{b}(0),\hat{c}(0))=(20,2,20\right)$, k(0)=0 and σ = 6. Two systems can achieve CMPS and the parameters were identified, as shown in Figure 4 and Figure 5.

## 5. Conclusions

We study the CMPS of FOCCS with unknown complex parameters, and propose a method for analyzing FOCCS without separating systems into real and imaginary parts. By this method, the constructed response system can be asymptotically synchronized to an uncertain drive system with a desired complex scaling diagonal matrix. The proposed synchronization scheme retains the complex nature of fractional-order complex chaotic systems. It not only provides a new method of analyzing FOCCS but also significantly decreases the complexity of computation and analysis. In future works, we will further investigate the synchronization of FOCCS, and generalize the obtained results to more general cases, i.e., FOCCS with time delay and external disturbances.
